# Suchilactone inhibits the growth of acute myeloid leukaemia by inactivating SHP2

**DOI:** 10.1080/13880209.2021.2017467

**Published:** 2021-12-28

**Authors:** Jingjing Wu, Yuan Deng, Xin Zhang, Jingjing Ma, Xinqi Zheng, Yue Chen

**Affiliations:** Department of Hematology, The Affiliated Huaian No.1 People's Hospital of Nanjing Medical University, Huai’an, China

**Keywords:** *Monsonia angustifolia*, ERK pathway proliferation apoptosis, network pharmacology

## Abstract

**Context:**

Suchilactone, a lignan compound extracted from *Monsonia angustifolia* E.Mey. ex A.Rich. (Geraniaceae), has little research on pharmacological activity; whether suchilactone has inhibitory effect on acute myeloid leukaemia (AML) is unclear.

**Objective:**

To investigate the antitumor effect of suchilactone and its mechanism in AML.

**Materials and methods:**

The effects of suchilactone on cell growth were detected by CCK-8 and flow cytometry. Network pharmacology was conducted to explore target of suchilactone. Gene expression was detected by western blot and RT-PCR. SHI-1 cells (1 × 10^6^ cell per mouse) were subcutaneously inoculated into the female SCID mice. Suchilactone (15 and 30 mg/kg) was dissolved in PBS with 0.5% carboxymethylcellulose sodium and administered (i.g.) to mice once a day for 19 days, while the control group received PBS with 0.5% carboxymethylcellulose sodium. Tumour tissues were stained with Ki-67 and TUNEL.

**Results:**

Suchilactone exerted an effective inhibition on the growth of SHI-1 cells with IC_50_ of 17.01 μM. Then, we found that suchilactone binds to the SHP2 protein and inhibits its activation, and suchilactone interacted with SHP2 to inhibit cell proliferation and promote cell apoptosis via blocking the activation of SHP2. Moreover, Suchilaction inhibited tumour growth of AML xenografts in mice, as the tumour weight decreased from 0.618 g (control) to 0.35 g (15 mg/kg) and 0.258 g (30 mg/kg). Suchilactone inhibited Ki-67 expression and increased TUNEL expression in tumour tissue.

**Discussion and conclusions:**

Our study is the first to demonstrate suchilactone inhibits AML growth, suggesting that suchilactone is a candidate drug for the treatment of AML.

## Introduction

Acute myeloid leukaemia (AML) is a clonal malignant proliferative disease of myeloid primordial cells in the haematopoietic system that can arise from the haematopoietic progenitor cells (Khwaja et al. 2016; Chopra and Bohlander 2019). The aetiology and pathogenesis of leukaemia is complex, and despite the recent progress, its aetiology is not fully understood (Khan et al. 2017). At present, chemotherapy is still the main treatment for AML; however, many patients with remission eventually relapse and develop refractory leukaemia, leading to treatment failure and death (Dombret and Itzykson 2017). According to recent surveys, the number of new leukaemia patients was 474 519, accounting for 2.5% of all cancers, and the number of deaths was 311 594, accounting for 3.1% of all cancers (Sung et al. 2021). At present, a variety of oral targeted drugs (Venetoclax, Glasdegib, and Dasatinib) have been for AML treatment (Paschka et al. 2018; Wolska-Washer and Robak 2019); however, they have limited efficacy and may induce significant toxicity (Ryan 2018). Therefore, there is an urgent need to find new drugs with fewer toxic effects for the treatment of AML. In cancer, from the 1940s to the end of 2014, approximately 49% of the 175 approved small molecules were discovered and developed from natural products or their novel structures (Newman and Cragg 2016).

**Figure 1.**
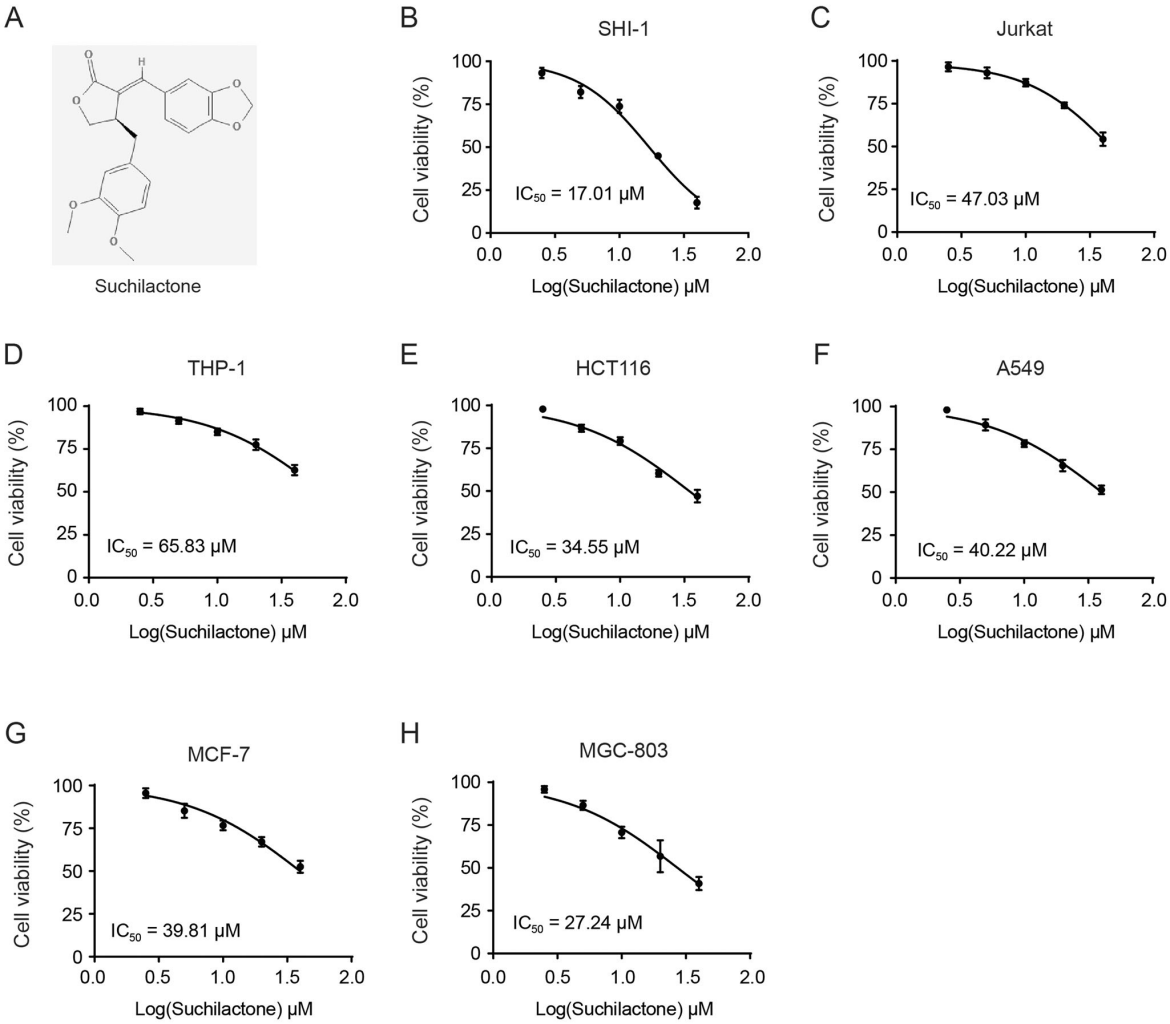
Effects of suchilactone on proliferation of various tumour cells. (A) The chemical structure of suchilactone. (B) SHI-1 cells, (C) Jurkat cells, (D) THP-1 cells, (E) HCT-116 cells, (F) A549 cells, (G) MCF-7 cells, and (H) MGC-803 cells were seeded in 96-well plates for 12 h and treated with different concentrations of suchilactone or 0.1% DMSO for 24 h. CCK8 solution was used to detect the cell viability at 450 nm on a full wavelength microplate reader, and the IC_50_ value calculated. The experiment was repeated three times.

Suchilactone (Figure 1(A)) is a natural product from *Monsonia angustifolia* E.Mey. ex A.Rich. (Geraniaceae), and is traditionally used as an indigenous vegetable for daily meals in Tanzania (Lyimo et al. 2003). *Monsonia angustifolia* appears to have pro-sexual stimulatory effects (Fouche et al. 2015). Recently, researchers found that several active components (justicidin A, 5-methoxyjusticidin A, suchilactone etc.) from *Monsonia angustifolia* could inhibit the production of Aβ, which in turn can ameliorate Alzheimer’s Disease (Chun et al. 2017). However, the role of suchilactone in other diseases has not been reported, so we have investigated its antitumor activity. After screening a variety of cell lines, we found that suchilactone has a good inhibitory activity on AML cells.

In this study, we aimed to elucidate the mechanism by which suchilactone inhibits the growth of AML. We found that suchilactone inhibited the proliferation and promoted the apoptosis of human SHI-1 cells. Suchilactone also significantly inhibited the tumour growth in Server Combined Immune-deficiency (SCID) mice. Finally, we report that suchilactone binds to the SHP2 (Protein Tyrosine Phosphatase Non-Receptor Type 11) protein and inhibits its activation, thereby suppressing the proliferation pathway and promoting the apoptosis pathway, which in turn inhibits the progression of AML.

## Materials and methods

### Cell culture and reagents

The human acute myeloid leukaemia cell line SHI-1, human T lymphocyte leukaemia cell line Jurkat, human colon cancer cell line HCT-116, human breast cancer cell line MCF-7, human lung cancer line A549, and human gastric carcinoma cell line MGC-803 were purchased from Guangzhou Saiku Biotechnology Co., Ltd. The SHI-1 cells were cultured in IMDM medium (Gibco, Grand Island, NY) containing 10% foetal bovine serum and 1% penicillin/streptomycin (Biological Industries, Cromwell, CT) at 37 °C in a 5% CO_2_ incubator. The Jurkat cells and THP-1 cells were cultured in RPMI1640 medium (Gibco, Grand Island, NY) containing 10% foetal bovine serum and 1% penicillin/streptomycin (Biological Industries, Cromwell, CT) at 37 °C in a 5% CO_2_ incubator. The HCT-116 cells, MCF-7 cells, A549 cells, and MGC-803 cells were cultured in BMDM medium (Gibco, Grand Island, NY) containing 10% foetal bovine serum and 1% penicillin/streptomycin (Biological Industries, Cromwell, CT) at 37 °C in a 5% CO_2_ incubator. 

Suchilactone (B51017) was purchased from Shanghai Yuanye Bio-Technology Co., Ltd. (Shanghai, China). TRIzol (RNAiso Plus, 9109) was purchased from TaKaRa Biotechnology (Dalian, China). The RNA reverse transcription kit (HiScript III RT SuperMix for qPCR, R323-01), SYBR Green Fluorescent Quantitative PCR Mix (ChamQ Universal SYBR qPCR Master Mix, Q711-02), CCK-8 Cell Counting Kit (A311-01), TUNEL FITC Apoptosis Kit (A111-01), and Annexin V-FITC/PI Apoptosis Detection Kit (A211) were purchased from Vazyme Biotech Co., Ltd. (Nanjing China). The anti-mouse/rabbit immunohistochemical detection kit was purchased from Proteintech (PK1006, Rosemont, IL, USA). Antibodies specific for BCL-2 (ab182858), BAX (ab32503), caspase-3 (ab32351), and actin (ab8227) were purchased from Abcam (Cambridge, UK). Antibodies specific for ERK (#4695), p-ERK (#4370) and Ki-67 (#9449) were purchased from Cell Signalling Technology (Danvers, MA, USA). Antibodies specific for SHP2 (sc-7384) and p-SHP2 (sc-293147) were purchased from Santa Cruz Biotechnology (Dallas, TX, USA).

### CCK-8 assay

The cytotoxicity of suchilactone against SHI-1 cells was assessed using Cell Counting Kit-8. Cells were seeded in 96-well plates (5 × 10^3^ cells/well) for 12 h and then treated with different concentrations of either suchilactone or 0.1% DMSO for 24 h. Then, 10 µL of CCK-8 solution was added to each well and incubated for 4 h. Finally, the absorption light of 450 nm was detected on a full wavelength microplate reader.

### Annexin V-FITC/PI apoptosis detection

SHI-1 cells were seeded in 12-well plates for 4 h and then treated with suchilactone or 0.1% DMSO for another 24 h. The cells were washed three times with cold PBS, and centrifuged at 1000 rpm for 5 min at 4 °C. The SHI-1 cells were then gently suspended in 100 µL binding buffer containing 5 µL Annexin V-FITC and 5 µL PI Staining Solution for 10 min in dark at room temperature. Then, 400 µL binding buffer was added, and the cells were analysed by flow cytometry (Invitrogen Attune NxT).

### SHP2 interferes lentivirus transfected SHI-1 cells

SHI-1 cells were seeded in 6-well plates for 12 h, then transfected with control or *SHP2* interferes lentivirus for 48 h. The sequences were 5′-TTCTCCGAACGTGTCACGT-3′ (shRNA-Ctrl), 5′-ACACTGGTGATTACTATGA-3′ (shRNA-*SHP2*).

### Real-time quantitative PCR (RT-PCR)

SHI-1 cells were seeded in 6-well plates for 4 h and then treated with suchilactone for another 24 h. The cells were washed three times with cold PBS, and centrifuged at 1,000 rpm for 5 min at 4 °C. Total RNA was extracted from SHI-1 cell using the TRIzol reagent, according to the manufacturer’s instructions. The reaction volume was 20 µL containing: 1 µg RNA, 5 µL 5× Hiscript III qRT SuperMix, and RNase Free dH_2_O. The cDNA was subjected to quantitative PCR, with a reaction volume of 10 µL with 1 µL cDNA, 5 µL qPCR mix, 0.75 µL 5 µM primers (Forward and Reverse), and 3.25 µL RNase-free dH_2_O. The primers were synthesized by GenScript Biotech Corporation (Nanjing, China) according to the following sequences:

GAPDH forward 5′-GGAGCGAGATCCCTCCAAAAT-3′
GAPDH reverse 5′-GGCTGTTGTCATACTTCTCATGG-3′
BCL2 forward 5′-GGTGGGGTCATGTGTGTGG-3′
BCL2 reverse 5′-CGGTTCAGGTACTCAGTCATCC-3′
BAX forward 5′-CCCGAGAGGTCTTTTTCCGAG-3′
BAX reverse 5′-CCAGCCCATGATGGTTCTGAT-3′

### Western blot

SHI-1 cells were seeded in 6-well plates for 4 h and then treated with suchilactone for another 24 h. The cells were washed three times with cold PBS, and centrifuged at 1,000 rpm for 5 min at 4 °C. The total protein of SHI-1 cell was lysed with WB-IP lysis containing 1% protease inhibitor for 30 min on ice. Total protein was assessed using a BCA protein quantitation kit. Protein samples were separated by 10–12% SDS-polyacrylamide gel electrophoresis and transferred to PVDF membranes at 350 mA for 90 min. The PVDF membranes were blocked with 5% BSA for 1 h, the strips with the indicated primary antibody overnight, and with the secondary antibody incubated for 90 min at room temperature. Finally, the strips were detected using a LumiGLO chemiluminescent substrate system (KPL, Gaithersburg, MD, USA).

### Network pharmacology technology

The PharmMapper database identifies potential targets for small molecules using the pharmacophore mapping method (Liu et al. 2010; Wang et al. 2016, 2017). The SDF file of suchilactone was obtained from the PubChem website and uploaded to the PharmMapper website, and 297 predicted targets were obtained. In addition, 374 targets related to AML were obtained from the DisGeNET database. We found 25 identical proteins in DisGeNET database and PharmMapper, and the possible 25 targets protein was selected by protein interaction analysis through the string website. Finally, KEGG pathway and GO analyses were performed on the common targets.

### Docking technology

The 3D structure of SHP2 was downloaded from the Protein Database (PDB ID: 5EHR), and the structures of suchilactone were downloaded from PubChem. The docking process was performed in Autodock4 with coarse docking using a simulated annealing algorithm and a subsequent refinement using a genetic algorithm, which were set to run 100 and 50 times, respectively.

### Cellular thermal shift assay (CETSA)

The SHI-1 cells were seeded in 10 cm plates for 12 h and then treated with suchilactone or 0.1% DMSO for another 4 h. The cells were collected, washed with cold PBS, and centrifuged at 1000 rpm for 5 min. The cells were then evenly divided into centrifugation tubes (70 µL each tube) and heated for 3 min at the following temperature: 46, 49, 52, 55, 58, 61, 64 and 67 °C, the samples were cooled for 3 min at temperature and then kept on ice. Then, the samples were placed in a refrigerator at −80 °C overnight. The samples were thawed at room temperature for 30 min and then refrigerated at −80 °C for 4 h. Finally, the samples were centrifuged at 13,000 rpm for 25 min, the supernatant added to the loading buffer, and analysed by western blotting.

### Transplantation tumour experiment

Female SCID mice (BALB/cJGpt-*Prkdc^em1Cd561^*/Gpt), 6–8 weeks old, were purchased from the Technology Centre of Gempharmatech (Nanjing, China). This study was approved by the ethics committee of Affiliated Huaian No.1 People’s Hospital of Nanjing Medical University and followed the arrive guidelines. The mice were reared in SPF grade animal room at 20 ± 2 °C under a 12 h light/dark cycle. The SHI-1 cells in the logarithmic growth phase were collected and centrifuged (1, 000 rpm, 5 min), resuspended in pre-cooled PBS, and the cell density adjusted to 1 × 10^7^/mL. Five days after the transplantation, mice were divided into control and suchilactone groups, with five mice in each group. Each mouse was subcutaneously inoculated in the armpit, with 1 × 10^6^ cells. The suchilactone group (15 and 30 mg/kg) was dissolved in PBS in 0.5% carboxymethylcellulose sodium was administered (i.g.) to mice once a day for 19 days, while the control group received PBS with only 0.5% carboxymethylcellulose sodium once a day for 19 days. The body weight and tumour volume of mice were recorded every other day, and the volume was calculated as (a × b^2^)/2, where ‘a’ represents the length of the tumour and ‘b’ represents the width of the tumour.

### Immunohistochemical staining

Paraffin sections of tumour tissue were immersed in xylene for 20 min to dewax, and then in 100%, 75% and 50% ethanol for 10 min. After the slices were subjected to antigen repaired with sodium citrate antigen repair solution, the endogenous hydrogen peroxide was inactivated with 3% hydrogen peroxide. After blocking with 5% goat serum for 1 h, the anti-Ki67 antibody (1:200) was added and incubated overnight at 4 °C. The anti-mouse/rabbit HRP labelled polymer (100 µL) was added and samples incubated at 37 °C for 30 min, followed by 100 µL of DAB working solution, and incubation at room temperature for 5 min. After 1 min of staining with haematoxylin, samples were washed in 50%, 75%, and 100% ethanol and xylene for 5 min, neutral gum was used to seal the film. The film was observed and photographed under a microscope.

### TUNEL staining

TUNEL assay was performed according to the manufacturer’s instructions. Paraffin sections of tumour tissue were immersed in xylene for 20 min to dewax, and then in 100%, 75% and 50% ethanol for 10 min. After treatment with proteinase K (20 mg/mL) for 30 min, the sections were stained with TUNEL-FITC (1: 200) and then counterstained with DAPI for 10 min. Images were acquired using fluorescence microscopy (IX61, Olympus, Tokyo, Japan).

### Statistical analysis

Statistical analysis was performed using GraphPad Prism software (version 7.0). All results are expressed as the mean ± SEM of three independent experiments. One-way ANOVA followed by Dunnett’s *post hoc* test was used to evaluate the differences when there were more than two groups. The Student’s *t*-test was used to evaluate the significant difference between the two groups. Statistical significance was set at *p* < 0.05.

## Results

### Suchilactone inhibited proliferation and promoted apoptosis of AML cells *in vitro*

**Figure 2.**
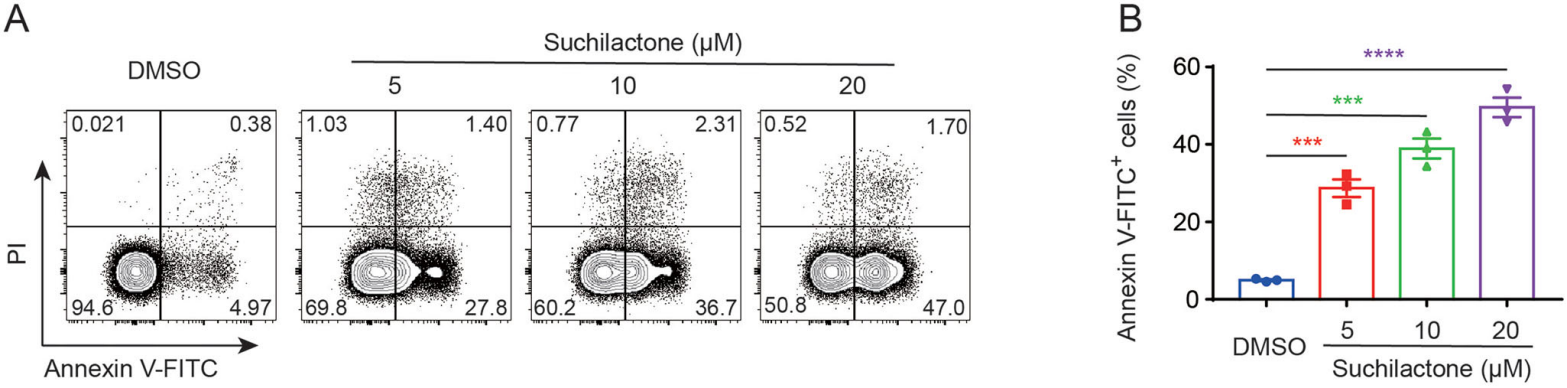
Suchilactone promoted apoptosis of SHI-1 cell *in vitro*. (A) SHI-1 cells were seeded in 12-well plates for 12 h and then treated with different concentrations of suchilactone or 0.1% DMSO for 24 h. The proportion of apoptotic cells were stained with Annexin V-FITC and PI, and then detected by flow cytometry. (B) The apoptosis rate of each group in (A) was quantitatively analysed. The experiment was repeated three times. Data are shown as the means ± SEM. ***p < 0.001, ****p < 0.00001 *vs.* the DMSO.

In order to investigate whether suchilactone has antitumor ability, we first selected a variety of tumour cell lines to evaluate the effects of suchilactone on their proliferation. We found that suchilactone inhibited the proliferation of AML cell line SHI-1 significantly better than Jurkat and THP-1, with the IC_50_ values of 17.01, 47.03, and 65.83 µM, respectively (Figure 1(B–D)). The inhibitory effect of suchilactone on the proliferation of colon cancer cell line HCT-116, lung cancer cell A549, breast cancer cell MCF-7 and gastric cancer cell MGC-803 was not superior to that of SHI1 cells, with the IC_50_ value of 34.53, 40.22, 39.81, and 27.24 µM, respectively (Figure 1(E–H)). From these results, we found that suchilactone has a better inhibitory effect on the proliferation of AML cell line SHI-1, then we will continue to explore its mechanism. Then, we treated SHI-1 cell with suchilactone for 24 h, flow cytometry showed that suchilactone induced apoptosis of SHI-1 cell, and that 20 µM suchilactone induced apoptosis in nearly 50% of the SHI-1 cells (Figure 2(A,B)). These results showed that suchilactone inhibited growth of AML cells by suppressing cell proliferation and inducing apoptosis.

### Network pharmacology analysis predicts potential molecular mechanisms of suchilactone in AML

**Figure 3.**
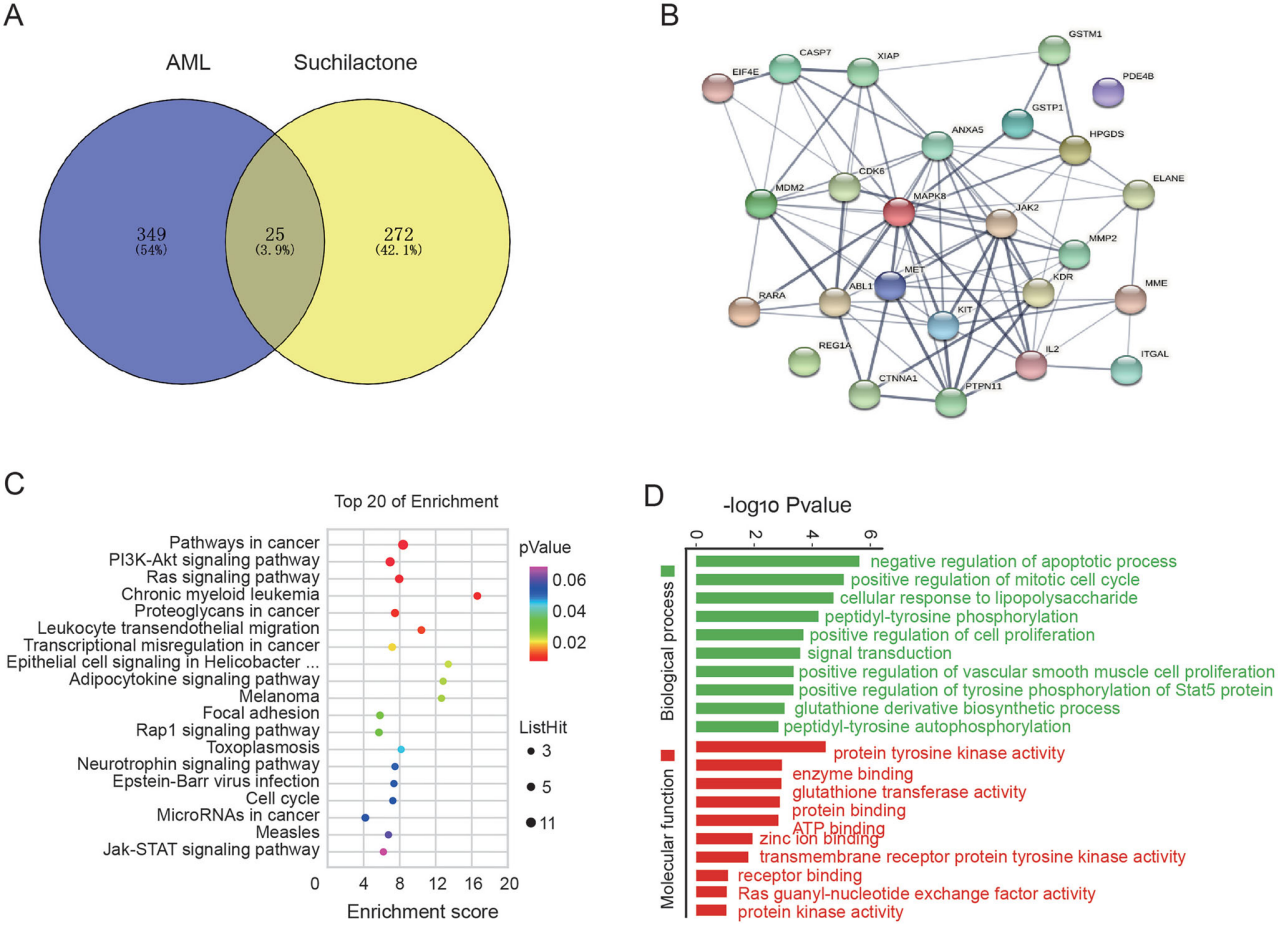
Network pharmacology analysis predicted the targets of suchilactone. The SDF file of suchilactone was obtained from the PubChem website and uploaded to the PharmMapper website; 297 predicted targets were obtained. Additional 374 targets related to acute myeloid leukaemia were obtained from the DisGeNET database. (A) Twenty-five common proteins of the two parts intersections were obtained and (B) the possible target protein was selected by protein interaction analysis using the string website. (C) KEGG pathway analysis and (D) GO analysis were performed on the obtained common targets.

Next, we further explore the mechanism of suchilactone regulating the growth of AML cells. We use network pharmacology to predict the possible mechanism and target protein. The SDF file of suchilactone was obtained from the PubChem website and uploaded to the PharmMapper website, and 297 predicted targets were obtained. In addition, 374 targets related to AML were obtained from the DisGeNET database. We found that 25 predicted proteins appeared in AML related DisGeNET database (Figure 3(A)), and the possible target proteins were selected through the protein interaction analysis on the string website (Figure 3(B)). From the interaction relationships, we observed SHP2(*PTPN11*), *JAK2* and MAPK8 to have significant advantages. The KEGG pathway analysis was also mainly enriched in malignant tumour, cell cycle, and Ras signalling pathways (Figure 3(C)). Moreover, GO analysis of the obtained protein revealed, that it might negatively regulate of apoptotic process and positively regulate cell proliferation (Figure 3(D)). Previous studies have report that SHP2 could regulate the proliferation and apoptosis of tumour cells, and that it plays an important role in tumour progression (Zhang et al. 2015; Pandey et al. 2017). Therefore, we speculated that SHP2 might be a candidate target protein of suchilactone in AML.

### Verification of interaction between suchilactone and SHP2

**Figure 4.**
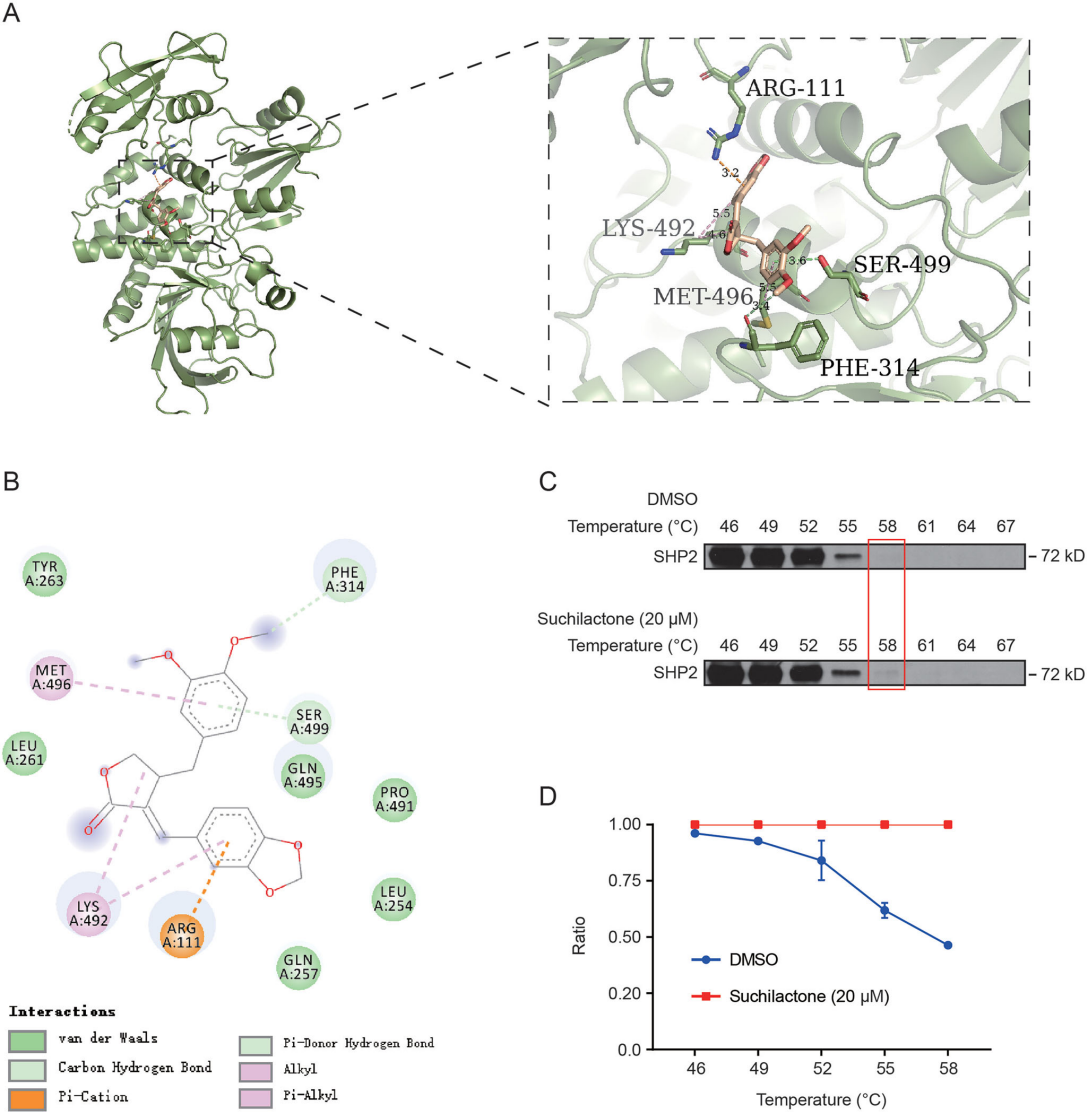
Verification of interaction between suchilactone and SHP2. The 3D structure of SHP2 was downloaded from PDB (PDB ID: 5EHR), and the structures of suchilactone were downloaded from PubChem. The docking process was conducted in Autodock4 with a coarse docking using a simulated annealing algorithm and a subsequent refinement using a genetic algorithm, which were set to run 100 and 50 times, respectively. (A) Structural diagram of interaction between suchilactone and SHP2. (B) Binding site of suchilactone on SHP2. The SHI-1 cells were seeded in 10 cm plates for 12 h and then treated with suchilactone or 0.1% DMSO for another 4 h. (C) CETSA was used to verify the interaction between suchilactone and SHP2. (D) The relative ratio of SHP2 was quantified between the two groups, the experiment was repeated three times.

To further explore the interaction between suchilactone and SHP2, we used *in silico* docking for molecular dynamics simulation between SHP2 (PDB ID: 5EHR) and suchilactone; we found that suchilactone interacted with SHP2 residues with ARG-111, PHE-314, LYS492, MET-496, and SER-499 (Figure 4(A,B)). CETSA was used to verify the binding of suchilactone and SHP2. Suchilactone treatment was found to be more stable than DMSO treatment at 55–58 °C (Figure 4(C,D)). The above data indicated a successful interaction of SHP2 and suchilactone.

### Suchilactone regulates the growth of AML cells by inhibiting the activation of SHP2

**Figure 5.**
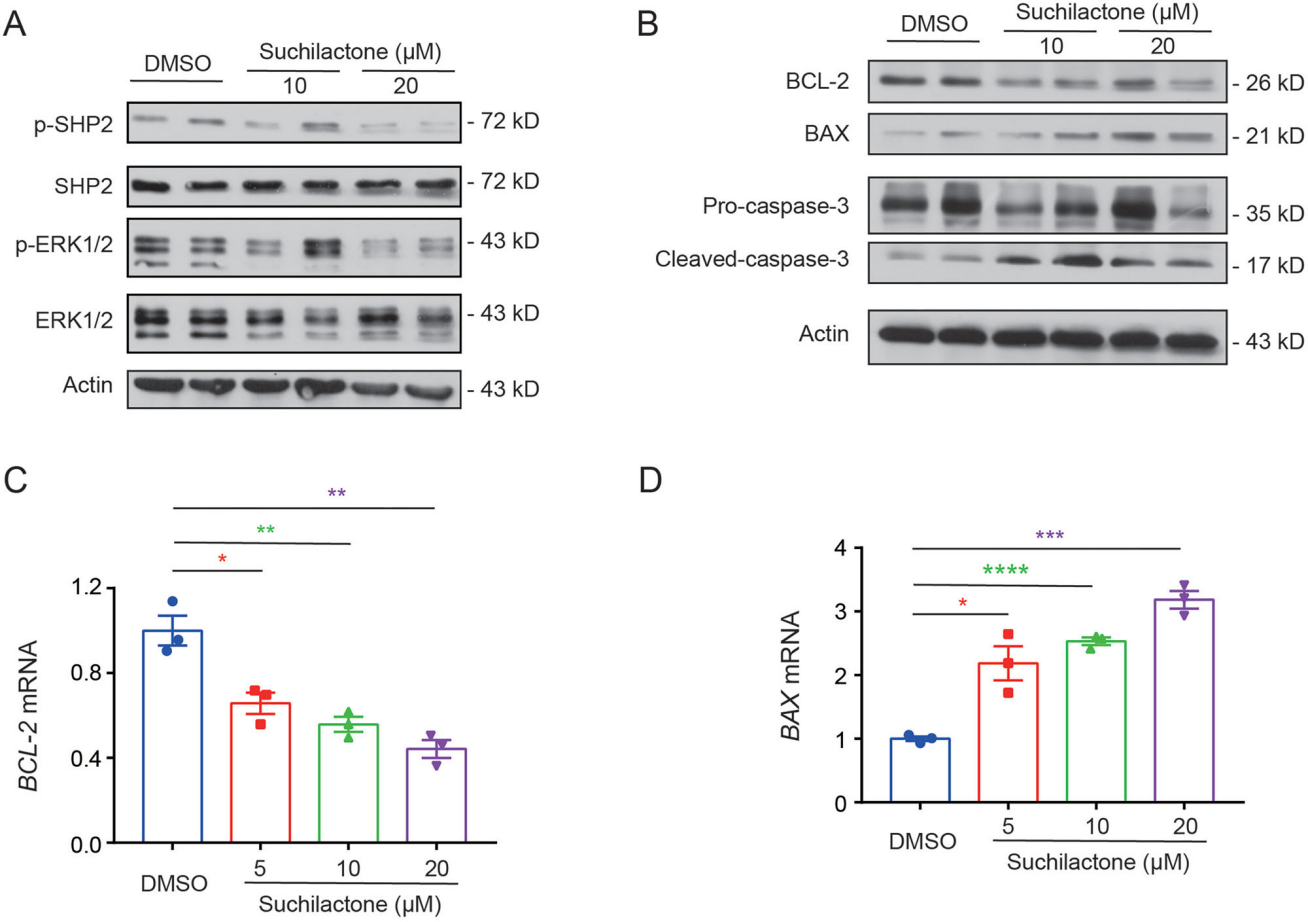
Suchilactone regulated the proliferation and apoptosis of AML cells by inhibiting the activation of SHP2. SHI-1 cells were seeded in 6-well plates for 12 h and then treated with different concentrations of suchilactone or 0.1% DMSO for 24 h. (A and B) Western blot was used to detect the activation of SHP2, ERK pathway, and apoptosis-related proteins. (C and D) Real-time quantitative PCR was used to detect the mRNA expression level of apoptosis-related genes. The experiment was repeated three times. Data are shown as the means ± SEM. *p < 0.05, **p < 0.01, ***p < 0.001, ****p < 0.00001 *vs.* the DMSO control.

Next, we explored how suchilactone affects SHP2 and inhibits the growth of AML cells. After treatment with suchilactone for 24 h, we found that suchilactone significantly inhibited the phosphorylation of SHP2 protein (Figure 5(A)), indicating that suchilactone could inhibit SHP2 activation. Studies have revealed that SHP2 can regulate the proliferation and apoptosis of tumour cells (Zhang et al. 2015; Pandey et al. 2017). Then, we detected the expression of proliferation and apoptosis pathway-related proteins. The results showed that suchilactone inhibited the phosphorylation of ERK and expression of BCL-2 (Figure 5(A)), and promoted the expression of BAX and caspase-3 (Figure 5(B)). Meanwhile, suchilactone also suppressed the mRNA expression of BCL-2 and induced the mRNA expression of BAX (Figure 5(C,D)).

**Figure 6.**
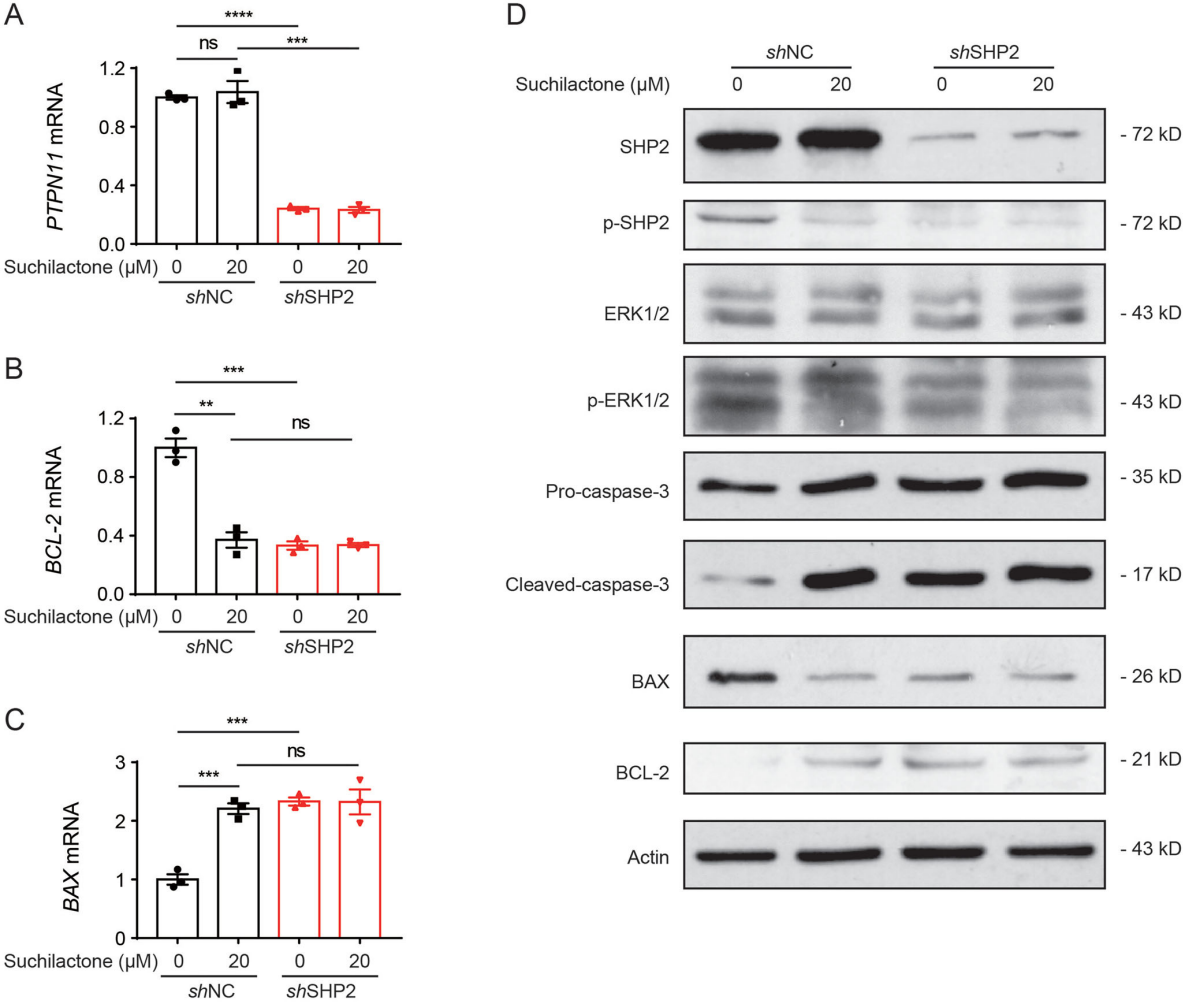
Knockdown of SHP2 blocked the effect of suchilactone on the growth of AML cells. SHI-1 cells were seeded in 6-well plates for 12 h, then transfected with control or *shSHP2* lentivirus for 48 h, finally, added different concentrations of suchilactone or 0.1% DMSO for 24 h. (A-C) Real-time quantitative PCR was used to detect the mRNA expression level of *SHP2*, *BCL-2*, and *BAX*. (D) Western blot was used to detect the activation of SHP2, ERK pathway, and apoptosis-related proteins. The experiment was repeated three times. Data are shown as the means ± SEM. *p < 0.05, **p < 0.01, ***p < 0.001, ****p < 0.00001.

In order to further verify that suchilactone inhibited the growth of AML cells depend on SHP2, we knockdowned SHP2 with lentivirus (Figure 6(A)). We found that that BCL-2 mRNA expression was down-regulated and BAX mRNA expression was up-regulated after SHP2 knockdown, and suchilactone did not significantly further regulate the expression of the two genes (Figure 6(B–C)). SHP2 suppression could decrease the expression of p-SHP2, p-ERK and BCL-2 and increase the expression of BAX and caspase-3, but suchilactone could not regulate this expression after SHP2 knockdown (Figure 6(D)). These results suggested that suchilactone inhibited proliferation and induced apoptosis of AML cells by blocking the activation of SHP2.

### Suchilactone inhibited the growth of xenograft tumours in AML

**Figure 7.**
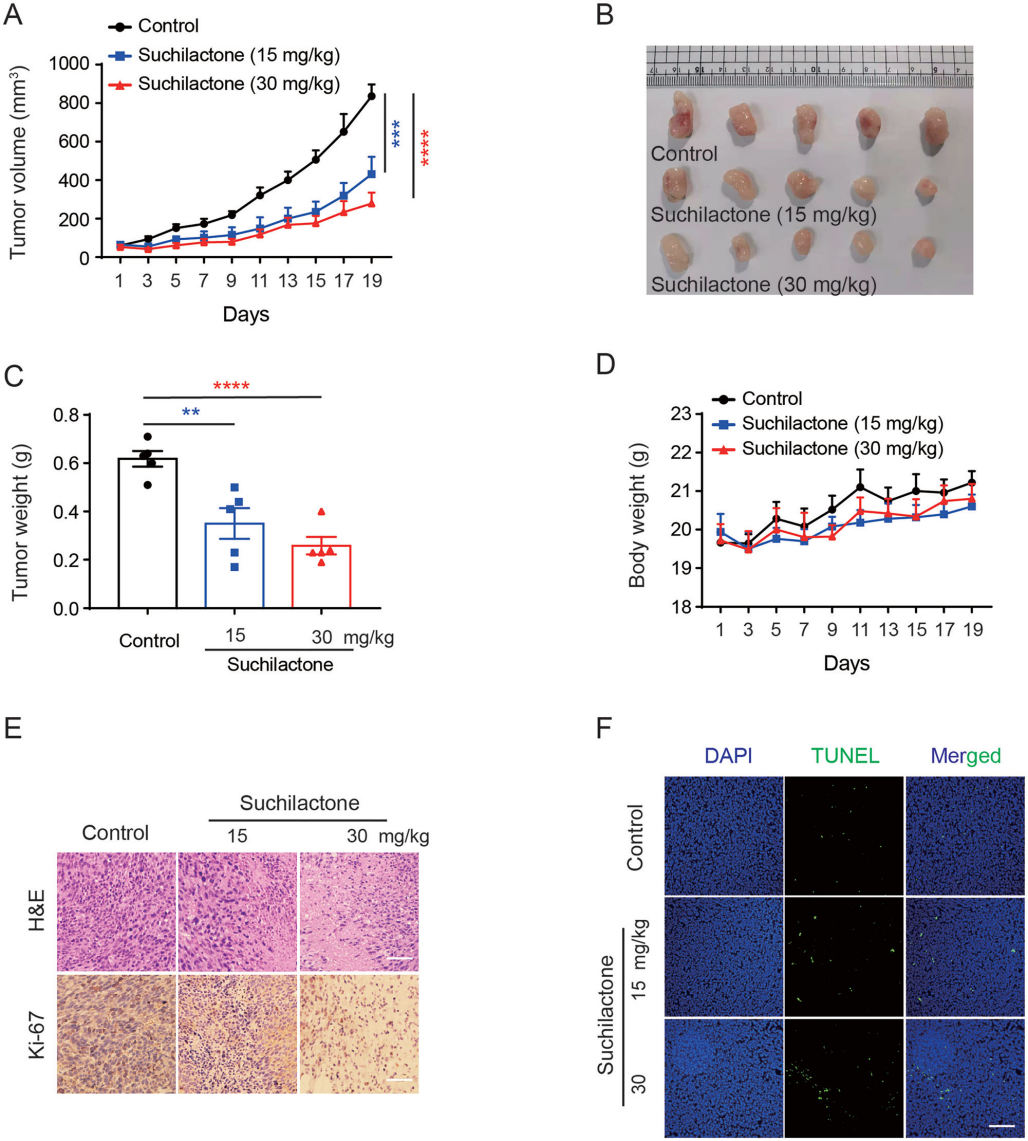
Suchilactone inhibited tumour growth of AML *in vivo*. SHI-1 cells were transplanted subcutaneously in SCID mice. Five days after transplantation, the mice were allocated to Control and suchilactone groups, with five mice in each group. Suchilactone (15 and 30 mg/kg) was administered once a day. (A) The tumour volume was measured every other day. After 19 days, the transplanted tumours were photographed (B) and weighed (C). (D) The weight of mice was measured every other day. (E) Paraffin sections of tumour tissues of mice were analysed by haematoxylin-eosin and Ki-67 staining. Scale bar: 50 µm. (F) The expression of TUNEL in tumour tissue sections was assessed by immunofluorescence staining. Scale bar: 50 µm. Data are shown as the means ± SEM. n = 5. *p < 0.05, **p < 0.01, ***p < 0.001, ****p < 0.00001 *vs.* the Control.

To further investigate whether suchilactone could inhibit the cell growth of AML *in vivo*, we inoculated SHI-1 cells under the skin of SCID mice to observe the xenograft tumour growth. Suchilactone could significantly inhibit tumour growth, and the tumour weight decreased from 0.618 g (control) to 0.35 g (15 mg/kg) and 0.258 g (30 mg/kg) (Figure 7(A–C)). Suchilactone had no effect on the body-weight of mice (Figure 7(D)), indicating no significant adverse effects. Haematoxylin-eosin (H&E) staining and Ki-67 staining showed that suchilactone inhibited tumour proliferation. Compared with the control group, suchilactone treatment group exhibited different tumour cell morphology and Ki-67 expression was also decreased (Figure 7(E)). Moreover, TUNEL staining showed enhanced green fluorescence after suchilactone treatment, indicating increased apoptotic tumour cells (Figure 7(F)). These data indicated that suchilactone inhibited the growth of AML cells *in vivo*.

## Discussion

AML is a highly heterogeneous hematological malignancy characterized by malignant proliferation of haematopoietic stem progenitor cells, and is one of the most common leukemias in adult hood (Malard and Mohty 2020). Recently, great progress has been made in the diagnosis and treatment of AML, and chemotherapy remains the primary treatment strategy for AML. New therapeutics including gemtuzumab ozogamicin, enascidini, midoseltalin, and novel liposome preparations of cytarabine and daunorubicin have recently been approved for AML (Luppi et al. 2018). At present, anthracycline combined with cytarabine is still the preferred induction therapy for young and middle-aged patients with AML. After induction therapy, a complete remission (CR) rate is 50–75%, and 30–40% of patients can achieve long-term survival (Tallman et al. 2005). However, 20–30% of young and 40–50% of elderly patients fail to undergo this treatment (Prebet et al. 2014). Moreover, the efficacy of these drugs is general, with a certain degree of toxicity. In recent years, Chimeric antigen receptor (CAR) T therapy has achieved good results in the treatment of AML (Gomes-Silva et al. 2019; Zhang et al. 2020), but the high cost makes it difficult for patients to bear. Therefore, there is an urgent need to develop novel therapeutics with high efficacy and low toxicity for the treatment of AML.

In recent years, with the development of traditional Chinese medicine, an increasing number of patients are adopting traditional Chinese medicine as an adjuvant treatment for cancer (Li et al. 2012; Nie et al. 2016). Researchers have found that patients with AML, through the treatment of traditional Chinese medicine, in addition to the standard treatment, may have a longer survival period (Bailly 2009; Pan et al. 2011). At the same time, some studies report that curcumin inhibits the growth and invasion of human monocytic leukaemia by altering the MAPK and MMP signalling pathways (Zhu et al. 2020) and that the ShengMaBieJia decoction (SMBJD) inhibits the angiogenesis in AML by acting on the PI3K/AKT pathway (Wang et al. 2020). Here, we reported that suchilactone, an active component from *Monsonia angustifolia*, could effectively inhibit the proliferation of the human AML cell line SHI-1 and promote apoptosis to alleviate the progression of AML in SCID mice.

Apoptosis, plays an important role in embryo formation, tissue development, homeostasis of internal environment, regulation of the immune system, and elimination of damaged, infected, and ageing cells (Perini et al. 2018). Studies have found that the BCL-2 anti-apoptotic protein is the main regulator of the BCL-2 protein family, and is the main apoptosis regulatory molecule related to cancer (Chanan-Khan 2004). BCL-2 protein can act on the mitochondrial outer membrane and affect mitochondrial outer membrane permeability, and preventing the release of cytochrome C to inhibit cell apoptosis (Kluck et al. 1997). BAX protein is a homolog of BCL-2, and is the main regulator of BCL-2 activity. BAX acts on the outer membrane of mitochondria to form ion channels, increases the permeability of the outer membrane of mitochondria, promotes the release of cytochrome C from mitochondria, activates the caspase cascade reaction, and promotes apoptosis (Zhang et al. 2017). BCL-2 protein expression upregulated in a variety of hematological malignancies (Grabow et al. 2016). We found that suchilactone significantly inhibited the expression of BCL-2 protein in SHI-1 cells, while promoting the expression of BAX and caspase-3 protein. These results indicated that suchilactone could inhibit the development of AML by regulating apoptosis signalling. However, the mechanism by which suchilactone regulates the apoptosis signalling pathway remains unclear.

SHP2 is a non-receptor protein tyrosine phosphatase encoded by the *PTPN11* gene, which is the first cloned phosphatase containing the SH2 domain (Feng et al. 1993). SHP2, as an intracellular response signal molecule of a variety of cytokines, growth factors and other extracellular stimuli, is widely expressed in various cells, and participates in important cellular activities, including cell proliferation, activation, migration, and differentiation (Tajan et al. 2015). Recent studies have found that SHP2 expression is increased in AML, and that the inhibition of SHP2 can improve the progression of acute leukaemia patients (Bentires-Alj et al. 2004; Richine et al. 2016). We found that suchilactone could bind to SHP2 and inhibit the activation of SHP2, thereby suppressing ERK-mediated cell proliferation. At the same time, we found that suchilactone could decrease the BCL-2 expression and increase the expression of BAX and caspase-3 in SHI-1 cells.

## Conclusions

This study is the first to confirm the effect of suchilactone on cell proliferation and apoptosis of AML, both *in vivo* and *in vitro*. Mechanically, we found that suchilactone suppressed the growth of AML cells by binding to the SHP2 protein and inhibiting its activation. Our data suggested that suchilactone might be a promising candidate for AML treatment.
